# Supplementary material for: Relationship between plasma uric acid levels, antioxidant capacity, and oxidative damage markers in overweight and obese adults: A cross-sectional study
Source: PLoS One. 2025 Jan 21;20(1):e0312217. doi: 10.1371/journal.pone.0312217 (PMC11750080; doi:10.1371/journal.pone.0312217)
Supplement: S1 Table — NW: Normal weight; OW: Overweight; O: Obese. The Chi-square test was applied to qualitative variables. The significance level was established at *p< 0.05. Data are presented as n (weighted %). (DOCX) [file pone.0312217.s001.docx]

**Table S1. Consumption frequency of food groups by participants**

| **Characteristic** | **NW**  **(n = 51)** | | **OW**  **(n = 27)** | | **O**  **(n = 15)** | | **p** |
| --- | --- | --- | --- | --- | --- | --- | --- |
| Fruit consumption, n (%) |  |  |  |  |  |  | 0.946 |
| 1 or more times per day | 12 | (25.50) | 6 | (24.00) | 4 | (26.70) |  |
| 1 or more times per week | 29 | (61.70) | 17 | (68.00) | 10 | (66.70) |  |
| Vegetable consumption, n (%) |  |  |  |  |  |  | 0.274 |
| 1 or more times per day | 14 | (29.80) | 6 | (23.10) | 3 | (20.00) |  |
| 1 or more times per week | 27 | (57.40) | 20 | (76.90) | 11 | (73.30) |  |
| White Fish consumption, n (%) |  |  |  |  |  |  | 0.034 |
| 1 or more times per week | 15 | (32.60) | 17 | (65.40) | 7 | (46.70) |  |
| 1–3 times per month | 27 | (58.70) | 7 | (26.90) | 5 | (33.30) |  |
| Oily Fish consumption, n (%) |  |  |  |  |  |  | 0.160 |
| 1 or more times per week | 12 | (26.70) | 19 | (34.60) | 4 | (30.80) |  |
| 1 – 3 times per month | 21 | (46.70) | 7 | (26.90) | 3 | (23.10) |  |
| Whole Dairy consumption, n (%) | | | | | | | 0.857 |
| 1 or more times per week | 20 | (41.70) | 11 | (42.30) | 7 | (46.70) |  |
| 1–3 times per month | 15 | (31.30) | 8 | (30.80) | 4 | (26.70) |  |
| Egg consumption, n (%) | | | | | | | 0.779 |
| 1 or more times per day | 7 | (15.20) | 5 | (19.20) | 2 | (13.30) |  |
| 1 or more times per week | 35 | (76.10) | 19 | (73.10) | 13 | (86.70) |  |
| Lean Meat consumption, n (%) | | | | | | | 0.287 |
| 1 or more times per day | 6 | (13.00) | 3 | (11.50) | 3 | (20.00) |  |
| 1 or more times per week | 29 | (63.00) | 19 | (73.10) | 10 | (66.70) |  |
| Fatty Meat consumption, n (%) | | | | | | | 0.864 |
| 1 or more times per week | 29 | (61.70) | 17 | (65.40) | 12 | (80.00) |  |
| 1–3 times per month | 14 | (29.80) | 6 | (23.10) | 2 | (13.30) |  |
| Legume consumption, n (%) | | | | | | | 0.371 |
| 1 or more times per day | 10 | (21.30) | 4 | (15.40) | 0 | (0.00) |  |
| 1 or more times per week | 33 | (70.20) | 21 | (80.80) | 15 | (100.0) |  |
| Nuts consumption, n (%) | | | | | | | 0.265 |
| 1 or more times per week | 14 | (29.80) | 14 | (53.80) | 8 | (53.30) |  |
| 1–3 times per month | 24 | (51.10) | 7 | (26.90) | 3 | (20.00) |  |
| Refined Cereals consumption, n (%) | | | | | | | 0.285 |
| 1 or more times per day | 18 | (38.30) | 5 | (19.20) | 3 | (20.00) |  |
| 1 or more times per week | 14 | (29.80) | 6 | (23.10) | 6 | (40.00) |  |
| Industrial Pastries consumption, n (%) | | | | | | | 0.207 |
| 1 or more times per week | 13 | (27.10) | 12 | (46.20) | 5 | (35.70) |  |
| 1–3 times per month | 25 | (52.10) | 10 | (38.50) | 4 | (28.60) |  |

NW: normal weight; OW: overweight; O: obese. The Chi-square test was applied to qualitative variables. Significance level was established at **p< 0.05*. Data are presented as n (weighted %).
