# Supplementary material for: Relationship between plasma uric acid levels, antioxidant capacity, and oxidative damage markers in overweight and obese adults: A cross-sectional study
Source: PLoS One. 2025 Jan 21;20(1):e0312217. doi: 10.1371/journal.pone.0312217 (PMC11750080; doi:10.1371/journal.pone.0312217)
Supplement: S2 Table — ** Indicates correlation is significant at p < 0.01. * Indicates correlation is significant at p < 0.05. Crude Model: No confounders were adjusted. Model I: Sex, level of education, alcohol consumption, tobacco use, physical activity, and age were adjusted. Model II: Sex, level of education, alcohol consumption, tobacco use, physical activity, age, and fruit, vegetable, white fish, and oily fish consumption were adjusted. (DOCX) [file pone.0312217.s002.docx]

**Table S2. Correlations between plasma uric acid and lipid profile**

| **Parameter** | | **Uric Acid (mg/dL)** | | |
| --- | --- | --- | --- | --- |
|  |  | **Crude Model** | **Model I** | **Model II** |
| BMI (kg/m^2^) | R | 0.447** | 0.427 | 0.367 |
|  | p |  | p = < 0.001 | p = 0.002 |
| Visceral Fat (%) | R | 0. 507** | 0.340 | 0.301 |
|  | p |  | p = 0.002 | p = 0.011 |
| Body fat (%) | R | 0.106 | 0.373 | 0.390 |
|  | p |  | p = < 0.001 | p = 0.001 |
| Total cholesterol (mg/dL) | R | 0.447** | 0.442 | 0.361 |
|  | p |  | p = < 0.001 | p = 0.002 |
| HDL cholesterol (mg/dL) | R | -0.408** | -0.436 | -0.337 |
|  | p |  | p = < 0.001 | p = 0.04 |
| LDL cholesterol (mg/dL) | R | 0.251* | 0.184 | 0.135 |
|  | p |  | p = 0.094 | p = 0.263 |
| Triglycerides (mg/dL) | R | 0.564** | 0.527 | 0.492 |
|  | p |  | p = < 0.001 | p = < 0.001 |
| Glucose (mg/dL) | R | -0.115 | -0.092 | 0.136 |
|  | p |  | p = < 0.001 | p = < 0.001 |
| Hba1c (%) | R | 0.047 | -0.070 | 0.003 |
|  | p |  | p = < 0.001 | p = < 0.001 |
| Creatinine (mg/dL) | R | 0.473** | 0.288 | 0.191 |
|  | p |  | p = < 0.001 | p = < 0.001 |

** Indicates correlation is significant at p < 0.01

* Indicates correlation is significant at p < 0.05

Crude Model: No confounders were adjusted.

Model I: Sex, level of education, alcohol consumption, tobacco use, physical activity, and age were adjusted.

Model II: Sex, level of education, alcohol consumption, tobacco use, physical activity, age, and fruit, vegetable, white fish, and oily fish consumption were adjusted.
